# 327. Assessment of Bacterial Co-infection Rates and Antibiotic Exposure in COVID-19 Patients

**DOI:** 10.1093/ofid/ofab466.529

**Published:** 2021-12-04

**Authors:** Adam J Luetkemeyer, Nick Bennett, Laura Aragon, Jeannette Ploetz, Sarah E Boyd

**Affiliations:** 1 Saint Luke’s Hospital, Kansas City, Missouri; 2 Saint Luke’s Health System, Kansas City, Missouri

## Abstract

**Background:**

COVID-19 pandemic data suggest risk for bacterial co-infection upon hospital presentation remain extremely low. Despite low co-infection rates, antibiotics are prescribed for most patients. Current data are limited regarding institutional-specific change in antibiotic use over the course of the pandemic. Given the low rates of co-infections, Saint Luke’s Health System’s COVID-19 Treatment Taskforce developed a COVID-19 evaluation and treatment order set which included procalcitonin (PCT) . As co-infection literature emerged, active education was provided, and order sets were modified to provide passive education regarding co-infection rates. We aimed to assess antibiotic practice changes as data and strategies to influence use evolved during the pandemic.

**Methods:**

This was a multi-center, single health-system retrospective cohort study. Ten community hospitals and 1 academic medical center were included in analysis. Inclusion criteria were age ≥18 years, admitted during April or September 2020 and had a positive COVID-19 result on admission. Patients were excluded if they were readmitted for COVID-19 related issues. Both primary and secondary outcomes were analyzed from the first 7 days after admission. The primary outcome was rate of respiratory bacterial co-infections. This was determined through sputum and blood cultures, urinary antigens including *Streptococcus pneumoniae* and Legionella, and PCT. Secondary outcomes included rate of antibiotic use, antibiotic days of therapy (DOT), length of therapy, and antibiotic use trends.

Baseline Characteristics

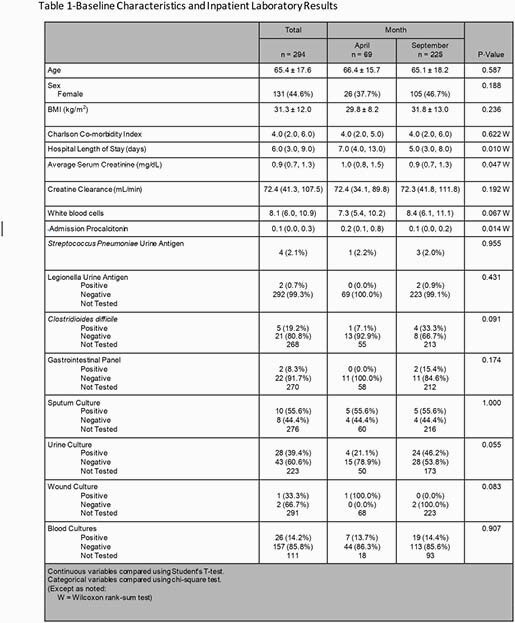

**Results:**

A total of 294 patients were included with 69 patients in April 2020 and 225 in September 2020. Primary and secondary results are shown in Table 2. Rate of culture-confirmed bacterial co-infection when examining April 2020 was 4.38% and 4.44 % in September 2020. Antibiotic uses, antibiotic DOT, and length of therapy were all significantly lower in September 2020 compared to April 2020.

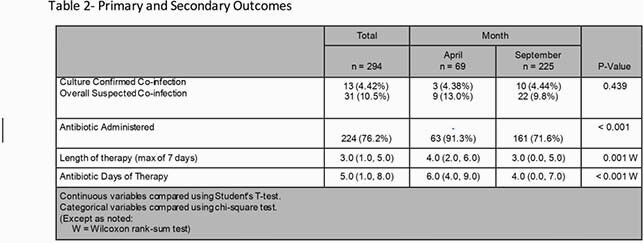

**Conclusion:**

Our results show bacterial co-infections were extremely low in our health system. Despite positive trends in antibiotic use, prescribing remained high. More targeted interventions to decrease antibiotic exposure in COVID-19 patients are needed.

**Disclosures:**

**All Authors**: No reported disclosures

